# B-Myb accelerates colorectal cancer progression through reciprocal feed-forward transactivation of E2F2

**DOI:** 10.1038/s41388-021-01961-9

**Published:** 2021-07-27

**Authors:** Xiaoyan Fan, Yitao Wang, Tinghui Jiang, Tao Liu, Yuelei Jin, Kailong Du, Yulong Niu, Chunxue Zhang, Zhongyu Liu, Yunlong Lei, Youquan Bu

**Affiliations:** 1 Department of Biochemistry and Molecular Biology, College of Basic Medical Sciences, Chongqing Medical University, Chongqing, 400016 China; 2 Molecular Medicine and Cancer Research Center, Chongqing Medical University, Chongqing, 400016 China; 3 Present Address: Dermopathic Research Institute, Taizhou University Hospital, Taizhou University, Taizhou, China

**Keywords:** Cancer genetics, Colorectal cancer

## Abstract

B-Myb is an important transcription factor that plays a critical role in gene expression regulation and tumorigenesis. However, its functional implication in colorectal cancer remains elusive. In this study, we found that B-Myb was significantly upregulated at both mRNA and protein levels in colorectal cancer samples compared to non-tumor counterparts. B-Myb overexpression accelerated cell proliferation, cell cycle progression and cell motility in colorectal cancer cells, and promoted tumor growth in orthotopic nude mouse models in vivo. In contrast, B-Myb depletion inhibited these malignant phenotypes. Mechanistic investigations revealed that E2F2 was a novel transcriptional target of B-Myb and is essential to B-Myb-induced malignant phenotypes. Notably, B-Myb and E2F2 exhibited positive expression correlation, and interacted with each other in colorectal cancer cells. In addition to their autoregulatory mechanisms, B-Myb and E2F2 can also directly transactivate each other, thus constituting consolidated reciprocal feed-forward transactivation loops. Moreover, both B-Myb and E2F2 are required for the activation of ERK and AKT signaling pathways in colorectal cancer cells. Taken together, our data clarified a critical role for B-Myb in colorectal cancer and unraveled an exquisite mutual collaboration and reciprocal cross regulation between B-Myb and E2F2 that contribute to the malignant progression of human colorectal cancer.

## Introduction

The colorectal cancer (CRC) is one of the most common cancers and the main cause of cancer-related deaths worldwide [1]. Although the dissemination of colonoscopy with polypectomy has led to a decline in the incidence and mortality rates, CRC is still the second most prevalent cancers among males and the third among females. For the patients diagnosed with stage I or II, 5-year relative survival rates are 91% and 82%, respectively. However, 5-year survival declines to 12% for stage IV disease [2]. The combination of surgical therapy and chemotherapy is one of the leading means to treat the colorectal tumors. However, the prognosis of patients with CRC remains relatively poor, with a recurrent rate over 30% after curative surgery [3, 4]. Thus, more efforts are needed to functionally identify novel CRC-related genes to develop novel therapeutic strategies for the treatment of CRC.

The myb gene family consists of three members, encoding three corresponding transcription factors of MYBL1 (A-Myb), MYBL2 (B-Myb), and MYB (c-Myb) with distinct biological functions [5–7]. A-Myb and c-Myb are restrictedly expressed in certain types of cells whereas B-Myb is found to be broadly expressed in all proliferating cells [8]. A-Myb plays an important function in spermatogenesis and mammary gland development [9], while c-Myb mainly controls the proliferation and differentiation of hematopoietic stem and progenitor cells [10]. Accordingly, A-MYB and c-Myb have been reported to be only involved in certain specific cancers, e.g., A-MYB in leukemia, and c-Myb in leukemia, colon, and breast cancer [5]. During the past decades, accumulating studies have focused on B-Myb, and have demonstrated that B-Myb regulates various biological processes including cell proliferation, cell differentiation, apoptosis, and is implicated in a broad spectrum of cancers [5–7]. B-Myb plays a crucial role on regulating cell cycle by interacting with other cell cycle regulators and transactivating downstream target genes, such as Cyclin B1 (CCNB1) and CDK1 to promote entry into the S- and M-phases of cell cycle [5]. In addition, several studies have reported that B-Myb is overexpressed and plays significant role in several types of cancers, such as breast cancer [6, 11], hepatocellular carcinoma [12], and renal cell carcinoma [13]. Our group has recently demonstrated that B-Myb is overexpressed and exerts a tumor-promoting role in non-small cell lung cancer [14, 15]. However, the functional involvement and the underlying molecular mechanisms of B-Myb in CRC have not yet been fully elucidated.

In this study, we demonstrated that the expression of B-Myb is frequently upregulated in CRC. We further discovered that E2F2 is a novel target gene of B-Myb, and B-Myb plays a tumor-promoting role in CRC via mutual collaboration and forming reciprocal feed-forward loops with E2F2, which is essential for the activation of ERK and AKT signaling pathways in CRC.

## Results

### Upregulation of B-Myb is associated with the progression of human colorectal cancer

**Fig. 1:**
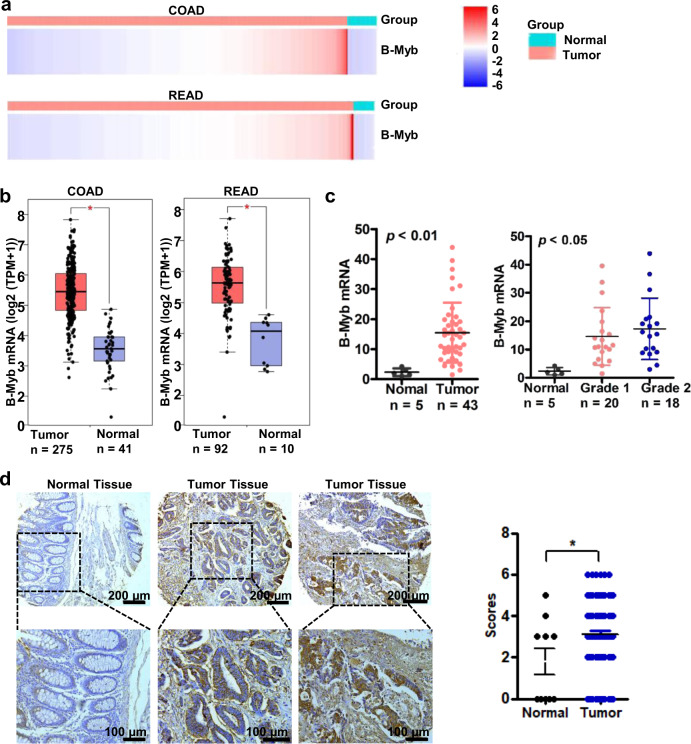
B-Myb is overexpressed in colorectal cancer. **a** Heatmap for B-Myb expression in colorectal cancer. B-Myb expression data were downloaded from the Cancer Genome Atlas (TCGA, https://portal.gdc.cancer.gov/). COAD Colon adenocarcinoma, READ rectum adenocarcinoma. **b** B-Myb mRNA overexpression in colorectal cancer. The TCGA B-Myb expression data were analyzed online by Gene Expression Profiling Interactive Analysis (GEPIA, http://gepia.cancer-pku.cn/). **c** Verification of B-Myb mRNA overexpression in colorectal cancer. Expression of B-Myb was determined using qRT-PCR with specific primers in colorectal cancer cDNA array purchased from OriGene (HCRT101). Grades: G1 and G2. **d** B-Myb protein overexpression in colorectal cancer. B-Myb protein expression was determined by immunohistochemical (IHC) analysis using colorectal cancer tissue microarray slides. Representative images (left) and quantification results (right) were indicated. *P < 0.05.

To examine the expression of B-Myb in human CRC, we first downloaded the B-Myb expression data of colon adenocarcinoma (COAD) and rectum adenocarcinoma (READ) from the Cancer Genome Atlas (https://portal.gdc.cancer.gov/). The heatmap data showed that the expression of B-Myb mRNA increased in both COAD and READ tissues (Fig. 1a). Quantitative analysis by Gene Expression Profiling Interactive Analysis (GEPIA, http://gepia.cancer-pku.cn/) confirmed the significant upregulation of B-Myb mRNA in CRC relative to normal colorectal tissues (Fig. 1b). Further quantitative RT-PCR (qRT-PCR) analysis on human CRC cDNA array (Supplementary Table S1) verified that B-Myb mRNA was indeed remarkably elevated in CRC samples compared with normal colorectal tissues (*p* < 0.01), and of note, the expression level of B-Myb mRNA was positively correlated with pathologic grade (*p* < 0.05, Fig. 1c). Furthermore, immunohistochemistry analysis on a colon cancer tissue microarray revealed that B-Myb was also significantly upregulated at the protein level in colon cancer samples compared with normal counterparts (Fig. 1d, Supplementary Table S2). Analysis of B-Myb expression in the context of various clinical pathologic features revealed that the expression level of B-Myb was positively correlated with pathologic grade (Supplementary Table S2). Collectively, these results suggest that B-Myb is a positive regulator for CRC progression and may serve as a potential diagnostic marker.

### B-Myb enhances colorectal cancer cell growth in vitro and in vivo

**Fig. 2:**
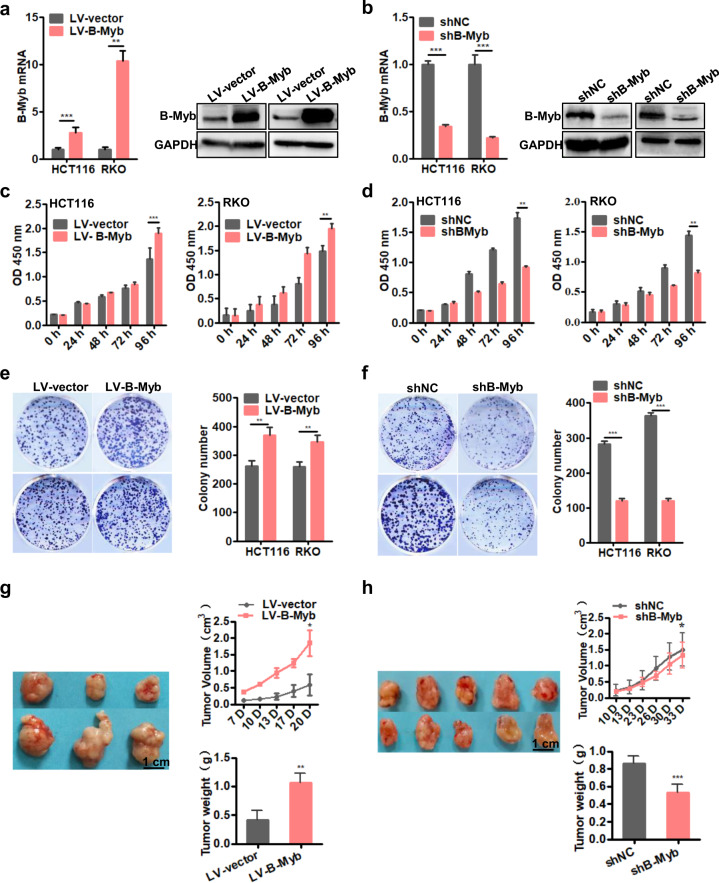
B-Myb enhances colorectal cancer cell proliferation. **a** Lentivirus-mediated stable B-Myb overexpression. HCT116 and RKO cells were infected with the empty control and B-Myb-expressing lentiviral particles, and then selected in the presence of puromycin to obtain the control (LV-control) and B-Myb overexpression (LV-B-Myb) stable cells. Expression of B-Myb was examined by qRT-PCR and immunoblot analysis. **b** Lentivirus-mediated stable B-Myb knockdown. HCT116 and RKO cells were infected with the lentiviral particles expressing negative control shRNA and B-Myb shRNA, and then selected in the presence of puromycin to generate the polyclonal control (shNC) and B-Myb knockdown (shB-Myb) stable cells. Expression of B-Myb was examined by qRT-PCR and immunoblot analysis. **c**, **d** B-Myb increases cell proliferation. Cell proliferation was detected by CCK8 assay in the stable B-Myb overexpression or knockdown cells at the indicated time points. **e**, **f** B-Myb increases colony formation. Cells were seeded on plastic plates for plate clone formation assay. Representative images were shown. **g**, **h** B-Myb promotes colorectal cancer growth in vivo. Stable B-Myb overexpression or knockdown HCT116 cells were injected subcutaneously into the dorsal flanks of nude mice. The tumor size was measured 1–2 times a week for tumor growth curve construction. The tumor weight was measured at the end of the experiment. Data represent the mean ± SD. All experiments were performed in triplicates. *p < 0.05, **p < 0.005, ***p < 0.001.

To assess the role of B-Myb in CRC progression, we used two different CRC cell lines, HCT116 and RKO, to generate stable B-Myb overexpression or knockdown cells. The overexpression or knockdown effects were verified by qRT-PCR and immunoblotting analyses (Fig. 2a, b). Next, we tested whether B-Myb could affect the proliferation in CRC cells by Cell Counting Kit-8 (CCK8) assays. The results showed that B-Myb overexpression led to enhanced proliferation in both HCT116 and RKO cells (Fig. 2c) while silencing B-Myb significantly inhibited proliferation in both HCT116 and RKO cells (Fig. 2d). Moreover, colony formation assays showed that overexpression of B-Myb in HCT116 and RKO cells significantly increased the number of colonies (Fig. 2e) while knockdown of B-Myb remarkably decreased colony-forming ability (Fig. 2f). These findings indicate that B-Myb promotes CRC cells growth in vitro. To extend our in vitro observations, we investigated whether B-Myb could regulate tumor growth of CRC cells in vivo. As shown in Fig. 2g, compared with the control group, B-Myb overexpression remarkably elevated tumor volume and tumor weight in nude mice, while B-Myb knockdown caused a significant inhibition of tumor volume and tumor weight (Fig. 2h), indicating that B-Myb can enhance colorectal tumor growth in vivo.

### B-Myb promotes cell cycle progression in colorectal cancer cells

**Fig. 3:**
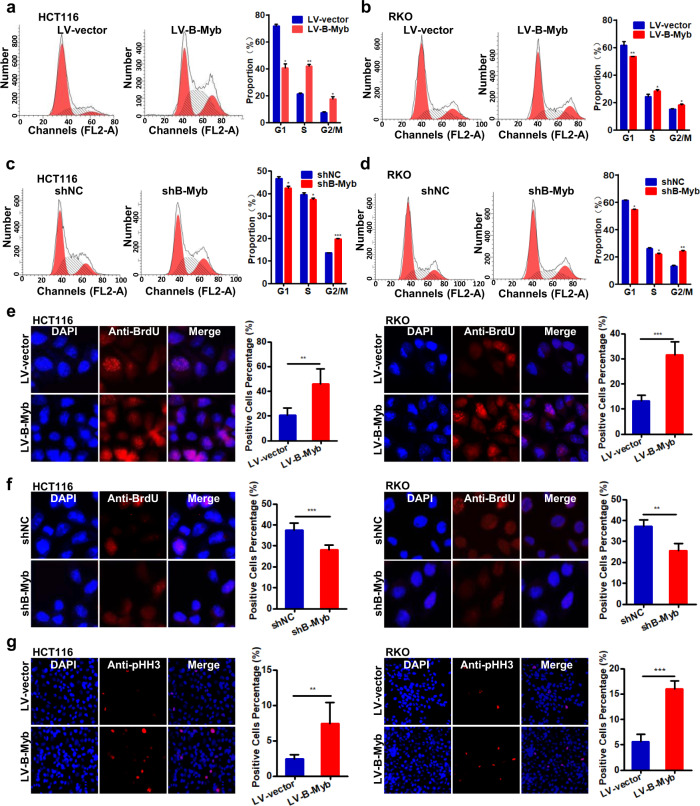
B-Myb promotes cell cycle progression in colorectal cancer cells. **a**–**d** Cell cycle distribution. The stable B-Myb overexpression or knockdown cells were seeded on six-well plates, and 24 h later, cells were collected and subjected to flow cytometer analysis. **e**, **f** BrdU labeling. DNA biosynthesis was labeled by BrdU (red), and DAPI was used to stain cell nuclei (blue) in the stable B-Myb overexpression or knockdown cells. BrdU positive cells were counted and subjected to statistical analysis. **g** The effect of B-Myb overexpression on mitosis. The stable B-Myb overexpression cells were fixed and stained with anti-phospho-histone H3 (pHH3) antibody (red). The pHH3-positive cells were counted and subjected to statistical analysis. Data represent the mean ± SD. All experiments were performed in triplicates. *p < 0.05, **p < 0.01, ***p < 0.001.

B-Myb is a key transcription factor controlling the progression into and through S and G2/M phases during cell cycle that fundamentally allows cell proliferation [5–7, 16]. Thus, we determined the effect of B-Myb on cell cycle progression in CRC cells by flow cytometry and indirect immunofluoresence. As shown in Fig. 3a, b, B-Myb overexpression resulted into remarkable progression into S and G2/M phase, as evidenced by the increased percentages of S and G2/M phase cells. Conversely, B-Myb knockdown delayed the progression into S phase and caused significant arrest in G2/M phase, as evidenced by the decreased percentage of S phase cells and increased percentage of G2/M phase cells (Fig. 3c, d). Consistently, BrdU labeling analysis revealed that B-Myb overexpression enhanced DNA biosynthesis in CRC cells (Fig. 3e), whereas B-Myb knockdown reduced DNA biosynthesis (Fig. 3f). In addition, phospho-histone H3 (pHH3) staining further confirmed that B-Myb overexpression caused a remarkable increase in the number of pHH3-positive mitotic cells as compared with that of control cells (Fig. 3g). Taken together, these data demonstrate that B-Myb promotes the cell cycle progression of CRC cells.

### B-Myb augments migration and invasion of colorectal cancer cells

**Fig. 4:**
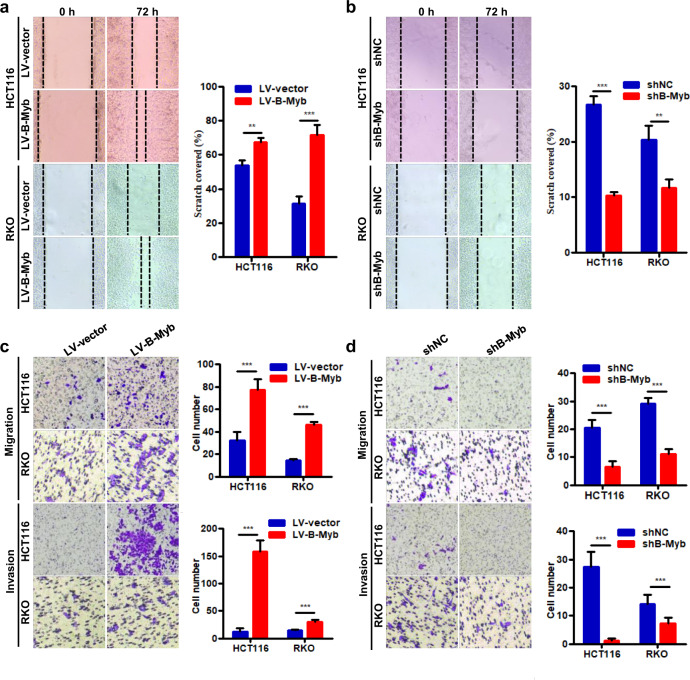
B-Myb augments migration and invasion in colorectal cancer cells. **a**, **b** Wound healing assay. The stable B-Myb overexpression or knockdown cells were subjected to wound healing assay. **c**, **d** Transwell migration and invasion assay. The stable B-Myb overexpression or knockdown cells were subjected to transwell migration and invasion assay, respectively. Representative images (×200) (left) and quantification results (right) were showed for each assay. Data represent the mean ± SD. All experiments were performed in triplicates. **p < 0.01, ***p < 0.001.

Furthermore, we investigated whether B-Myb affected migration and invasion of CRC cells in vitro. Wound healing assay showed that B-Myb overexpression induced a significantly increased rate of wound closure in both HCT116 and RKO cells (Fig. 4a). Consistently, B-Myb knockdown obviously suppressed lateral migration ability compared with control group (Fig. 4b). Moreover, B-Myb overexpression increased the migration and invasion while B-Myb knockdown significantly reduced that in both HCT116 and RKO cells as demonstrated by transwell migration and matrigel invasion assays, respectively (Fig. 4c, d). These results indicate that B-Myb positively regulates the migration and invasion of CRC cells.

### Identification of signaling pathways and oncogenic signatures correlated with B-Myb

**Fig. 5:**
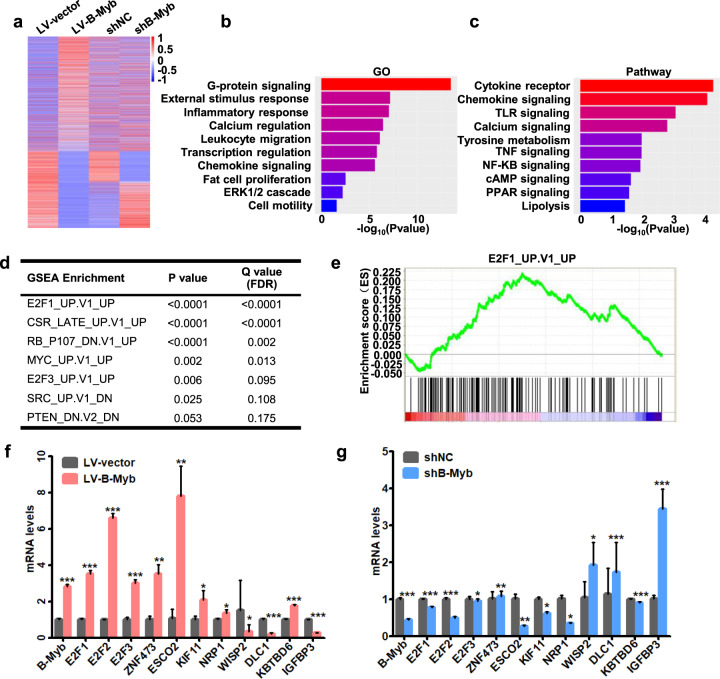
Identification of signaling pathways and oncogenic signatures correlated with B-Myb. **a** RNA-seq analyses for stable B-Myb overexpression and knockdown HCT116 cells. Heatmap shows the differentially expressed genes. **b**, **c** GO enrichment and KEGG pathway analysis based on the differentially expressed genes between the stable B-Myb overexpression (LV-B-Myb) and its control (LV-vector) HCT116 cells. **d** Top enriched gene sets by GSEA analysis. False discovery rate, FDR. **e** GSEA plot showing that the B-Myb-regulated genes correlate with E2F1 gene signatures (E2F1_UP.V1_UP). **f**, **g** Verification of important B-Myb-regulated downstream genes by qRT-PCR. Data represent the mean ± SD. *p < 0.05, **p < 0.01, ***p < 0.001.

To unravel the potential molecular mechanism underlying B-Myb-promoted malignant phenotype in CRC cells, we conducted RNA-seq analysis to determine the gene expression profile changes at whole genomic level in stable B-Myb overexpression and knockdown cells. We found 3520 genes that were commonly dysregulated in response to B-Myb overexpression and knockdown (Fig. 5a). Gene ontology (GO) analysis showed that the differentially expressed genes were enriched in cell proliferation, cell motility, transcription regulation, ERK1/2 cascade etc (Fig. 5b and Supplementary Fig. 1a). Pathway analysis showed that the dysregulated genes are involved in multiple cancer-related pathways, such as TNF signaling, NF-κB signaling, and c-AMP signaling, etc (Fig. 5c and Supplementary Fig. 1b). Moreover, gene set enrichment analysis (GSEA) analysis using Molecular Signature Database gene sets yielded several cancer-related gene sets, including E2F1-activated gene signatures (E2F1_UP.V1_UP), serum-stimulated gene signatures (CSR_LATE_UP.V1_UP), MYC-activated gene signatures (MYC_UP.V1_UP), etc (Fig. 5d, e and Supplementary Fig. 2). We further confirmed a series of key downstream genes of B-Myb in these significantly affected pathways and gene sets by qRT-PCR analysis, including E2F1, E2F2, E2F3, ZNF473, ESCO2, KIF11, NRP1, WISP2, DLC1, KBTBD6, and IGFBP3 (Fig. 5f, g). These results strongly suggest that B-Myb might transactivate a second wave of transcription cascade of E2F family especially E2F2 which showed the most significant changes among E2F1, E2F2, and E2F3 in response to B-Myb overexpression and knockdown.

### B-Myb and E2F2 mutually regulate the transcription of each other

**Fig. 6:**
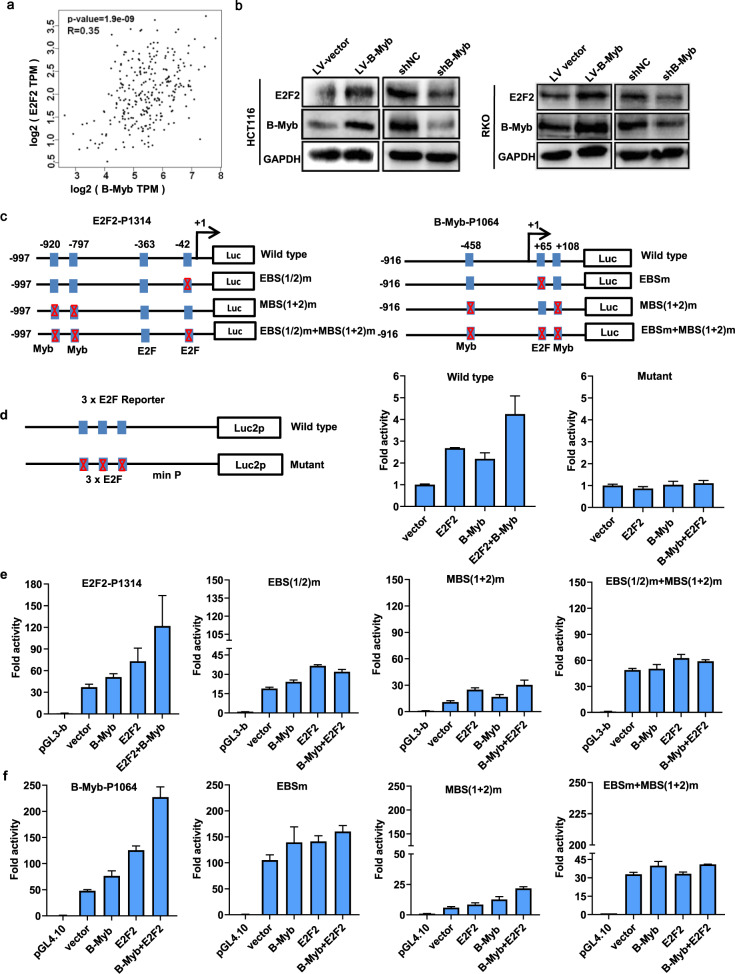
Reciprocal regulation between E2F2 and B-Myb. **a** Pearson correlation analysis of B-Myb and E2F2 in colorectal cancer samples (*n* = 177). **b** B-Myb upregulates E2F2 expression. Immunoblotting was performed to determine B-Myb and E2F2 expression in stable B-Myb overexpression and knockdown cells. **c** Schematic illustration of the wild-type and mutant E2F2-P1314 and B-Myb-P1064 luciferase (Luc) promoter reporter. The transcription start sites for E2F2 and B-Myb genes are indicated as +1. The E2F and Myb-binding sites are shown as boxes. The mutated E2F binding sites (EBS) and/or Myb-binding sites (MBS) are crossed. **d** Schematic illustration of the wild-type and mutant 3xE2F reporter and luciferase reporter assay. **e**, **f** Luciferase reporter assays. HCT116 cells were transiently transfected with the indicated plasmids, and 48 h after transfection, the luciferase activities were measured as described in “Materials and Methods” section. Data are expressed as fold change normalized to the activity of cells transfected with the empty pGL3-basic or pGL4.10 promoter-less vector alone (relative value, 1.0).

To further explore the regulatory mechanism between B-Myb and E2F2, we first examined the correlation between the B-Myb and E2F2 mRNA expression in CRC samples using the GEPIA database. The results showed that B-Myb expression was highly correlated with E2F2 expression in CRC (Fig. 6a). Consistent with the observations at mRNA level (Fig. 5f), immunoblotting showed that B-Myb positively regulates E2F2 protein expression, suggesting that E2F2 is a novel target gene of B-Myb (Fig. 6b). Transcription factor binding sites analysis further revealed that human E2F2 gene promoter contains consensus binding sites for both E2F and B-Myb, and luciferase reporter assay demonstrated that overexpression of E2F and/or B-Myb could transactivate the promoter activity of E2F2 gene. Accordingly, mutations of the E2F and/or Myb-binding sites significantly abolished the transactivated promoter activity driven by E2F2 and/or B-Myb. These data strongly indicate that the transcription of E2F2 could be regulated by B-Myb as well as E2F2 itself (Fig. 6c, e). In addition, overexpression of E2F2 and/or B-Myb could also enhance the luciferase activities of wild-type E2F reporter but not the mutant E2F reporter, suggesting their general transactivation effects on E2F gene families including E2F1-3 (Fig. 6d). Notably, human B-Myb gene promoter also harbors consensus binding sites for both E2F and B-Myb, and overexpression of E2F and/or B-Myb could also transactivate the promoter activity of B-Myb gene. Again, mutations of the E2F and/or Myb-binding sites remarkably abolished the transactivated promoter activity driven by E2F2 and/or B-Myb, strongly indicating that the transcription of B-Myb could be regulated by E2F2 as well as B-Myb itself (Fig. 6c, f). Together, these results suggest that B-Myb and E2F2 mutually regulate the transcription of each other.

### B-Myb interacts with E2F2 in colorectal cancer cells

**Fig. 7:**
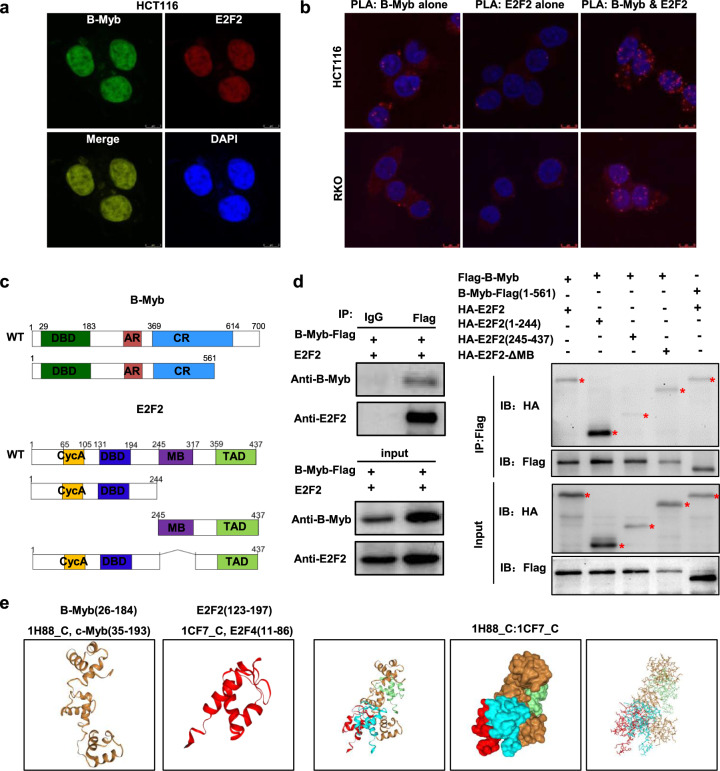
Interaction between E2F2 and B-Myb. **a** Colocalization of B-Myb and E2F2 protein expression in cell nuclei. HCT116 cells were co-transfected with LV203-B-Myb-Flag and pcDNA3.0-E2F2 expression vector. Forty-eight hours after the transfection, cells were fixed and stained with anti-B-Myb antibody (green) and anti-E2F2 antibody (red). Scale bar 5 μm. **b** Proximity ligation assays (PLAs) of B-Myb with E2F2 in HCT116 and RKO cells. Red spots are regions of signal amplification. Nuclear stain (Hoechst) is blue. Incubation with either B-Myb or E2F2 antibody alone was used as a control. Scale Bars: 10 μm. **c** Schematic illustration of the B-Myb and E2F2 structure and deletion mutants. DBD DNA-binding domain, AR acidic region/transactivation domain, CR conserved region, Cyc A Cyclin A/CDK2 binding domain, MB marked box, TAD Transactivation domain. **d** B-Myb associates with E2F2. HCT116 cells were co-transfected with LV203-B-Myb-Flag and pcDNA3.0-E2F2 expression vector (left panel). HEK293 cells were transiently co-transfected with LV105-Flag-B-Myb or pCDH-puro-HA-E2F2 with the indicated deletion mutants. Forty-eight hours after the transfection, whole cell lysates were prepared and subjected to co-immunoprecipatation assay. **e** Predicted models of B-Myb-E2F2 protein docking. The 3D homologous structures of B-Myb and E2F2 were searched by online HDOCK server, and the three-dimensional homologous docking models for B-Myb and E2F2 interaction were predicted.

The mutual regulation between B-Myb and E2F2 promoted us to further investigate their potential interaction. Firstly, immunofluorescence (IF) staining showed that B-Myb co-localized with E2F2 in cell nuclei (Fig. 7a). Then, we used in situ proximity ligation assay (PLA), an assay that can visualize protein interaction in close proximity (<40 nm) in cells. As shown in Fig. 7b, fluorescent PLA spots were easily detected when using antibodies against both B-Myb and E2F2, indicating that these two proteins are located in close proximity (<40 nm) and interacted with each other in vivo. To further determine whether B-Myb could associate with E2F2 in vivo, cells were transiently co-transfected with the expression plasmids for both B-Myb and E2F2. The co-immunoprecipitation assay demonstrated that anti-B-Myb-Flag immunoprecipitates contained E2F2, suggesting that B-Myb indeed forms a complex with E2F2 in vivo (Fig. 7c, d). To our surprise, in our attempt to perform in vitro GST pull-down assay, we found that B-Myb and E2F2 GST fusion proteins could not successfully expressed in bacterial cells probably due to their high toxicity and/or low stability of the exogenously expressed fusion proteins (data not shown). We thus turned to subsequent co-immunoprecipitation assay using serial truncated mutants (Fig. 7c). Of note, two C-terminal truncated mutants of B-Myb-Flag (210–700 AA and 360–700 AA) did not show obvious expression in cells (data not shown). Nevertheless, the results revealed that the amino acid 1–244 region of E2F2 showed strong capability to bind B-Myb, and the amino acid 1–561 region of B-Myb alone could bind to E2F2 (Fig. 7d). In support of this notion, we further performed a computational docking analysis of B-Myb and E2F2 through the HDOCK server. The docking analysis revealed three top-scored homologous docking models of interaction between N-terminal region of B-Myb and N-terminal region of E2F2 (Fig. 7e). Taken together, these results suggest the interaction of B-Myb and E2F2 is probably mediated at least by their N-terminal regions.

### E2F2 is required for B-Myb-induced malignant phenotypes

**Fig. 8:**
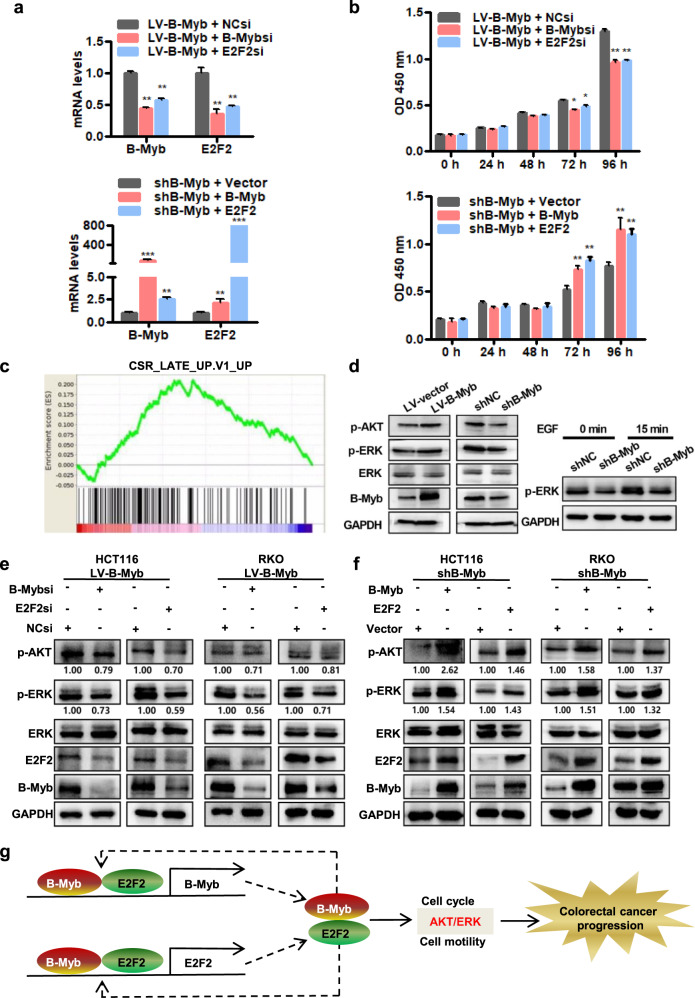
B-Myb and E2F2 participate in activation of ERK and AKT pathways. **a** siRNA mediated silencing of B-Myb and E2F2. Stable B-Myb overexpression (LV-B-Myb, upper panel) HCT116 cells were transiently transfected with negative control siRNA, B-Myb siRNA, or E2F2 siRNA. Stable B-Myb knockdown (shB-Myb, lower panel) HCT116 cells were transiently transfected with pcDNA3.0 empty vector, LV203-B-Myb-Flag and pcDNA3.0-E2F2 expression constructs. Twenty-four hours after transfection, qRT-PCR was performed to determine B-Myb and E2F2 expression. **b** E2F2 is required for B-Myb-induced cell proliferation. Cells were transiently transfected as described in (**a**), and cell proliferation was detected by CCK8 assay at the indicated time points. **c** GSEA plot showing that the B-Myb-regulated genes correlate with CSR_LATE gene signatures (CSR_LATE_UP.V1_UP). **d** B-Myb is essential to activation of ERK and AKT pathways. Immunoblotting was performed to determine B-Myb, ERK, p-ERK, and p-AKT expression in the stable B-Myb overexpression (LV-B-Myb) and its control (LV-vector) HCT116 cells (Left). Stable B-Myb knockdown (shB-Myb) and its control (shNC) HCT116 cells were subjected to serum starvation for 2 h, and then treated with EGF (200 ng/mL) for 15 min. Immunoblotting was performed to determine p-ERK expression (Right). **e** E2F2 is required for B-Myb-induced activation of ERK and AKT pathways. Stable B-Myb overexpression (LV-B-Myb) HCT116 and RKO cells were transiently transfected with negative control (NC) siRNA, B-Myb siRNA and E2F2 siRNA, respectively. Forty-eight hours later, cells were subjected to immunoblotting with the indicated antibodies. The band intensity ratios of pAKT or pERK to GAPDH are shown below the bands. **f** B-Myb and E2F2 activate ERK and AKT pathways. Stable B-Myb knockdown (shB-Myb) HCT116 and RKO cells were transfected with pcDNA3.0 empty vector, LV203-B-Myb-Flag and pcDNA3.0-E2F2 expression constructs, respectively. Forty-eight hours later, cells were subjected to immunoblotting with the indicated antibodies. **g** Work model. B-Myb and E2F2 regulate the transcription of each other (reciprocal feed-forward regulation) as well as their own transcription (autoregulation). In addition, B-Myb and E2F2 associate with each other (collaboration) and regulate various downstream target genes to activate ERK and AKT pathways and induce the malignant cell phenotype in colorectal cancer. Data represent the mean ± SD. All experiments were performed in triplicates. *p < 0.05, **p < 0.01, ***p < 0.001.

We next sought to determine the role of E2F2 in the B-Myb-induced malignant phenotypes in CRC cells. Intriguingly, in the stable B-Myb-overexpressing HCT116 or RKO cells, silencing of either B-Myb or E2F2 significantly repressed the expression of both B-Myb and E2F2 mRNAs (Fig. 8a; Supplementary Fig. 3a). Conversely, in the stable B-Myb-knockdown HCT116 or RKO cells, overexpression of either B-Myb or E2F2 remarkably enhanced the expression of both B-Myb and E2F2 mRNAs (Fig. 8a; Supplementary Fig. 3a). These data further supported the autoregulation and reciprocal regulation of B-Myb and E2F2. Furthermore, CCK8 assay showed that silencing of either B-Myb or E2F2 inhibited B-Myb-induced proliferation in the stable B-Myb-overexpressing HCT116 or RKO cells whereas overexpression of either B-Myb or E2F2 could restore B-Myb shRNA-inhibited proliferation in the stable B-Myb-knockdown HCT116 or RKO cells (Fig. 8b; Supplementary Fig. 3b). Moreover, wound healing and transwell migration assays showed that silencing of either B-Myb or E2F2 could inhibit B-Myb-promoted migration in the stable B-Myb-overexpressing HCT116 or RKO cells (Supplementary Figs. 3c, d and 4a, b), while overexpression of either B-Myb or E2F2 could restore B-Myb shRNA-mediated inhibition of migration in the stable B-Myb-knockdown HCT116 or RKO cells (Supplementary Figs. 3c, d and 4a, b). These data indicate that E2F2 is required for B-Myb-induced proliferation and motility in CRC cells.

### E2F2 is essential for B-Myb-mediated activation of ERK and AKT pathways

Our GSEA analysis yielded several remarkable growth-related and oncogene-related gene sets, such as CSR_LATE_UP.V1_UP, E2F1_UP.V1_UP, and MYC_UP.V1_UP (Figs. 8c, 5d, e and Supplementary Fig. 2), which are intimately implicated in ERK and AKT pathways. In addition, we recently reported that B-Myb can promote the activation of ERK and Akt signaling pathways through the inhibition of IGFBP3 in lung cancer [15]. Consistently, our RNA-seq and qRT-PCR demonstrated that IGFBP3 expression was also significantly repressed by B-Myb in CRC cells (Fig. 5f, g). Therefore, we finally sought to examine whether B-Myb could regulate ERK and AKT pathway to exert its tumor-promoting effects. As shown in Fig. 8d, B-Myb overexpression increased phosphorylation of both AKT and ERK, while B-Myb knockdown reduced phosphorylation of both AKT and ERK in HCT116 cells (Fig. 8d). Notably, B-Myb knockdown also attenuated the phosphorylation of ERK induced by EGF treatment (Fig. 8d), suggesting that B-Myb is a positive regulator for the activation of ERK and AKT pathways in CRC cells. Moreover, silencing of either B-Myb or E2F2 repressed the phosphorylation of AKT and ERK in the stable B-Myb-overexpressing HCT116 or RKO cells (Fig. 8e), whereas overexpression of either B-Myb or E2F2 increased the phosphorylation of AKT and ERK in the stable B-Myb-knockdown HCT116 or RKO cells (Fig. 8f). Taken together, these results indicate that E2F2 is essential for B-Myb-mediated activation of ERK and AKT signaling pathways in CRC cells, and also suggest that B-Myb may mediate activation of ERK and AKT pathways at least through upregulation of IGFBP3 in CRC cells.

## Discussion

### B-Myb is a pivotal tumor-promoter in colorectal cancer

Previous studies have shown that B-Myb, as a key transcription factor, plays an important role in regulating cell cycle progression and tumorigenesis. Nearly 30 years ago, Sala et al. firstly reported that constitutive expression of human B-Myb induced a transformed phenotype accompanied by activation of cyclin D1 and cdc2 expression [17]. However, the role of B-Myb in tumorigenesis has been only deeply explored in a few types of cancers. Thorner et al. reported that high B-Myb expression correlates with poor outcomes and pathological complete response to neoadjuvant chemotherapy in breast cancer [11]. Calvisi et al. demonstrated that B-Myb is upregulated during for hepatocellular carcinoma development, and high B-Myb expression serves as poorer prognostic marker [18]. In the present study, we found that the expression of B-Myb was significantly elevated in CRC, and high expression of B-Myb was significantly associated with tumor grade and size. Our functional experiments demonstrated that B-Myb overexpression significantly induced CRC cell proliferation and cell motility accompanied by cell cycle progression, whereas B-Myb knockdown inhibits the malignant phenotypes. The in vitro growth-promoting effects were further verified in xenograft nude mouse models. All the results suggest that B-Myb essentially contributes to CRC progression by promoting cell cycle progression, cell proliferation, and migration. Out present study is in accordance with previous preliminary observations describing the potential link between B-Myb and colon cancer [19–21].

### Exquisite mutual collaboration and cross regulation between B-Myb and E2F2

Currently, B-Myb has been proposed to exert its biological functions through two main mechanisms [5, 6]. First, B-Myb directly binds to the promoters and transactivates downstream target genes regulating cell cycle regulation (e.g., CCNB1, CDK1), cell survival (e.g., Myc), and invasion (e.g., SNAI1) [5]. Second, B-Myb can directly interact with some proteins to regulate cell phenotypes. For example, the most well characterized functions of B-Myb in cell cycle progression are mediated by its interaction with the DREAM complex. B-Myb is also shown to directly interact with the serine–threonine kinase receptor-associated protein, a kinase critical for TGF-β and p53 signaling pathways, to regulate cell survival [5, 6].

In this study, our mechanistic investigation revealed an exquisite mutual collaboration and cross regulation between B-Myb and E2F2. Feed-forward loop regulation is an effective and widespread strategy of transcriptional control program in both prokaryotes and metazoans [22, 23]. Feed-forward loops render cells resistant to transient inductive signals and thereby reduce spurious cellular phenotype alterations, help stabilize and enforce robust differentiation programs as well as cell phenotype [22, 23]. Here, we showed that B-Myb and E2F2 forms reciprocal feed-forward loops that function prominently to maintain the activation of AKT and ERK signaling pathways and malignant phenotype in CRCs cells (Fig. 8g). Of note, our results also revealed that either B-Myb or E2F2 can transactivate its own promoter, thereby forming dual positive autoregulatory loops. In addition, we also demonstrated that B-Myb and E2F2 bind to each other in cells. Therefore, the autoregulatory and interaction mechanisms are of potential biomedical significance as they could further consolidate the effectiveness, stability, and robustness of reciprocal feed-forward loops of B-Myb and E2F2. Conceivably and reasonably, targeting the reiterative feed-forward control system of B-Myb and E2F2 would be a promising therapeutic strategy for the treatment of CRCs, which is currently under investigation in our lab.

Previous studies reported that E2Fs including E2F2 and B-Myb have been shown to be coordinated by the DREAM complex, and E2Fs mainly regulate gene expression in G1/S and B-Myb regulates gene expression in S and G2/M phases during cell cycle [5–7]. However, our data and other previous studies showed that E2Fs and B-Myb also regulate multiple events/phases throughout cell cycle progression, as well as other biological processes, such as invasion, apoptosis and cell senescence in a cell cycle independent manner [20, 21]. Therefore, our study also suggests that E2F2 and B-Myb collaboration and mutual regulation are of broad biological significance which warrants further deep investigations.

### Potential oncogenic role of E2F2 in colorectal cancer

E2F2 belongs to the E2F family of transcription factors which generally divided into the two groups, i.e, “activators” (E2F1-3) and “repressors” (E2F4-8) [24, 25]. Along with E2F1 and E2F3, E2F2 has been well documented to have an essential role in the control of cellular proliferation and oncogene-mediated transformation [26–28]. Overexpression of E2F1 and E2F3 has been reported to be overexpressed and have a tumor-promoting effect in many cancers including lung cancer, breast cancer, and ovarian cancer [24, 29–32]. However, the research on the role of E2F2 in cancers is very limited. E2F2 overexpression has been reported in cancers, such as ovarian cancer [24, 32]. Of note, E2F2 transgenic mice showed high incidence of thymic epithelial tumors [33]. Reimer et al. reported that among E2F family members, especially E2F2 plays a pivotal role in ovarian cancer and might be candidates for specific therapeutic target [32]. However, the role of E2F2 in CRC remains largely elusive [34, 35]. In the present study, our data revealed that among E2F1-3, the expression of E2F2 responded most significantly to B-Myb, and E2F2 is also essential for B-Myb-mediated malignant phenotype and ERK/AKT activation. These data highly suggest that compared with E2F1 and E2F3, E2F2 might specifically play a pivotal role in CRC and thus serve as a specific therapeutic target. Therefore, further studies are needed to deeply investigate the function and molecular mechanisms of E2F2 in details in CRCs.

In conclusion, our study revealed that B-Myb is overexpressed in CRC, and transactivates and interacts with E2F2 to promote human CRC cell proliferation, cell cycle progression, motility, at least in part, through activation of ERK and AKT pathways. We additionally demonstrated that B-Myb and E2F2 forms reciprocal feed-forward loops that function prominently to maintain the malignant phenotype in CRCs cells (Fig. 8g). Taken together, our research suggests that B-Myb is a vital tumor-promoter in CRC.

## Materials and methods

### Cell culture

Human CRC cell lines, HCT116 and RKO, were obtained from Chinese Academy of Sciences Shanghai cell bank (Shanghai, China). Cells were routinely maintained in DMEM (Hyclone, Utah, USA) and MEM (Hyclone) medium supplemented with 10% of fetal bovine serum (Hyclone), penicillin (10^7^ U/L), and streptomycin (10 mg/L) in a humidified incubator containing 5% CO_2_ at 37 °C. Cell lines were tested by short tandem repeat analysis and tested for mycoplasma, and the last time of authentication for these cells was April 2019 from the supplementary file of our previous study [36].

### Tissue microarrays and Immunohistochemistry

B-Myb protein expression was determined on a CRC tissue microarray slide from AurageneBioscience Corporation (TC0167, Hunan China). Results were assessed by two independent experienced pathologists who were blinded to the experiment separately. The CRC tissue microarray TC0167 contains human CRC (110 cases) and adjacent normal colorectal tissues (10 cases). The detailed clinical information of tissue microarray was provided in Supplementary Table S3. Immunohistochemical analysis was conducted as described previously [37]. The information of the antibodies used was provided in Supplementary Table S4.

### qRT-PCR and immunoblotting analysis

Total RNA isolated from colon cancer cells using the Total RNA Kit I (Omega Bio-Tek). qRT-PCR was carried out by using the SYBR® Premix Ex Taq (Perfect Real Time, TAKARA) as described previously [2]. TissueScan cDNA Array for CRC (HCRT101) was purchased from OriGene Technologies (Rockville, MD, USA), and the clinical pathological information was provided in Supplementary Table S1. Immunoblotting analysis was performed with minor modifications as described previously [14, 37]. The information of the primers and antibodies used was provided in Supplementary Table S4 and Supplementary Table S5.

### Stable B-Myb overexpression and knockdown cell generation

The lentiviral empty vector (EX-NEG-LV203) and human B-Myb expression vector (EX-B0073-LV203) with a C-terminal Flag tag were purchased from GeneCopoeia (Guangzhou, China), and used to produce recombinant lentiviral particles as described previously [14, 37]. Lentiviral (GV248) particles carrying negative control and B-Myb shRNA were purchased from Genechem (Shanghai, China). Forty-eight hours after lentivirus infection, cells were selected in the presence of puromycin (HCT116 1 μg/ ml, RKO 1 μg/ ml) for about 2 weeks to generate the stable B-Myb overexpression and knockdown cells [14, 37].

### Cell proliferation, migration, and invasion assays

Cell proliferation was measured using CCK8 (Dojindo, Tokyo, Japan) [14, 37]. Would healing, transwell migration, and invasion assays were performed as described previously [14, 37].

### FACS analysis

Cells were collected by trypsin digestion and low-speed centrifugation, and then subjected to cell cycle distribution analysis on a FACScan flow cytometer as described previously [14, 37].

### RNA-sequencing analysis

For RNA-seq analysis, the exponentially grown B-Myb stable overexpression or silencing cells as well as its corresponding control cells were collected, total RNA was extracted, and cDNA libraries were then constructed and sequenced as described previously [14]. Differentially expressed genes were subjected to GO Enrichment Analysis and Kyoto Encyclopedia of Genes and Genomes Pathway analyses. GSEA (http://www.broad.mit.edu/gsea) was conducted to identify the significantly changed pathways and oncogenic signatures.

### Indirect immunofluorescence (IF)

The indirect immunofluorescent assays were conducted as described previously with minor modifications [38]. Briefly, after seeding and transfection, cells were sequentially fixed, permeabilized, and blocked. Cells were then incubated with the primary antibody, followed by the incubation with the second antibody. Cells were finally mounted onto glass slides and visualized with confocal microscope. The nucleus was stained with medium containing 4′,6-diamidino-2-phenylindole (Vector Laboratories), and the newly systhesized DNA was labeled by BrdU. The antibodies used were listed in Supplementary Table S4.

### Luciferase reporter assays, recombinant constructs, and site-directed mutagenesis

The promoter regions of E2F2 (−984/+329) and B-Myb (−916/+148) were obtained by PCR-based amplification and cloned into pGL3-basic and pGL4.10 vector to generate reporters, E2F2-P1314 and B-Myb-P1064, respectively. The three tandem consensus E2F binding sites were cloned into pGL4.27 [luc2P/minP/Hygro] to generate the 3 × E2F reporter. A series of luciferase reporter mutants harboring mutations in E2F and/or Myb-binding sites have been constructed by the site-directed mutagenesis kit (TOYOBO, Osaka, Japan) based on the three parental constructs of E2F2-P1314, B-Myb-P1064 and 3 × E2F reporter as described previously [39]. The pCDH-puro-HA-E2F2 expression plasmids with N terminal HA tag were constructed by amplifying the entire coding region of human E2F2 from pcDNA3.0-E2F2 (a generous gift from Prof. Chuangui Wang) and subcloning into pCDH-CMV-MCS-EF1-Puro. The sequences of primers used are listed in Supplementary Table S6. All the constructs were validated by direct sequencing. For luciferase reporter assay, cells were seeded in triplicate into 12-well plates and co-transfected with the corresponding plasmids, and luciferase activities were measured using Dual-Luciferase assay system (Promega) as described previously [39].

### Additional materials and methods

The details of other materials and methods are provided in the Supplementary materials and methods.

## Supplementary information

Supplementary Materials and methods

Supplementary Tables

Supplementary Figure Legends

Supplementary Figure 1-4
