# Supplementary material for: B-Myb accelerates colorectal cancer progression through reciprocal feed-forward transactivation of E2F2
Source: Oncogene. 2021 Jul 27;40(37):5613–25. doi: 10.1038/s41388-021-01961-9 (PMC8445821; doi:10.1038/s41388-021-01961-9)
Supplement: Supplementary file 1 — Supplementary Materials and methods [file 41388_2021_1961_MOESM1_ESM.pdf]

Supplementary materials and methods

Tumor xenografts

Cells were subcutaneously injected into the dorsal flank of four to six-week old nude mice (BALB/c). All nude mice were randomly divided into different groups (n = 3 or 5). Tumor size and tumor weight were measured and calculated as described previously[1]. All experimental procedures involving animals were performed in accordance with animal protocols approved by Laboratory Animal Center of Chongqing Medical University.

Proximity ligation assay (PLA)

PLA was performed using the Duolink in situ PLA Kit (Sigma) according to the manufacturer's instructions. PLA signals, recognized as red fluorescent dots, were visualized and images were taken by confocal microscopy (Leica TCS SP8).

Co-immunoprecipitation (co-IP) assay

Cells were lysed with IP lysis buffer, and protein concentration was determined routinely. Whole cell extracts were precleared with Protein A/G Magnetic Beads (Bimake) for 1 h at 4°C, and then incubated with primary anti-Flag antibody (Sigma) and normal rabbit IgG (Beyotime) overnight at 4°C. Protein A/G Magnetic Beads were then added to capture the antigen-antibody complexes for 1 h at room temperature. Magbeaded immunoprecipitates were then seperated on Magnetic separator, eluted out by boiling in SDS sample buffer, and finally processed for immunoblotting with the indicated antibodies. LV105-Flag-B-Myb and pCDH-puro-HA-E2F2 expression plasmids with a N-terminal Flag or HA tag were

constructed by PCR using LV105-B-Myb (purchased from FulenGen) or pcDNA3.0-E2F2 as the templates. The truncated constructs of pCDH-puro-HA-E2F2(1-244), pCDH-puro-HA-E2F2(245-437) and pCDH-puro-HA-E2F2△MB were generated by PCR using pCDH-puro-HA-E2F2 as the template. The deletion construct of LV203-B-Myb-Flag(1-561) was generated by PCR using LV203-B-Myb as the template.

Protein-protein docking analysis

The 3D homologous structures of B-Myb and E2F2 were predicted by RCSB Protein Data Bank online tool (http://www.rcsb.org/pdb/home/home.do). Protein-protein docking analysis was carried out using the HDOCK server (http://hdock.phys.hust.edu.cn/)[2,3]. Three top models were provided regarding possible interaction between B-Myb and E2F2[4].

siRNA transfection

The negative control small interfering RNA (siRNA), and siRNAs against B-Myb and E2F2 were chemically synthesized by GenePharma (Shanghai, China). The sequences of the siRNAs used were provided in Supplementary Table S6. The siRNAs were transfected into cells at a final concentration of 10 nmol/L using Lipofectamine RNAiMAX reagent (Invitrogen) according to the manufacturer's instructions.

Bioinformatics and database

The Cancer Genome Atlas (TCGA) colorectal cancer RNA-seq data were downloaded from TCGA data portal (https://cancergenome.nih.gov/). The expression levels B-Myb were assessed by either analyzing the TCGA transcriptomic data or retrieving

Gene Expression Profiling Interactive Analysis (GEPIA) online[5]. Heat maps were used to visualize the difference of gene expression using the pheatmap package of R3.6.2. Correlation analysis between B-Myb and E2F2 expression level was conducted by Pearson using the GEPIA database.

Statistical analysis

Statistical analyses were carried out using the SPSS 16.0 statistical software package (SPSS Inc, Chicago, USA). Qualitative variables were compared using Chi-square tests whereas quantitative variables were analyzed by Student's t-test. The distribution of data was expressed as mean ± SD. The difference between different groups was evaluated by Student’s t tests or Mann–Whitney U tests. Data were analyzed using the GraphPad Prism software version 5.0 for Windows (GraphPad software, San Diego California USA), and P values less than 0.05 were considered to be statistically significant.

References

[1] Jin Y, Zhu H, Cai W, Fan X, Wang Y, Niu Y, et al. B-Myb Is Up-Regulated and Promotes Cell Growth and Motility in Non-Small Cell Lung Cancer. Int J Mol Sci. 2017; 18: 860.

[2] Yan Y, Tao H, He J, Huang S-Y. The HDOCK server for integrated protein-protein docking. Nat Protoc. 2020; 15: 1829-1852.

[3] Yan Y, Zhang D, Zhou P, Li B, Huang S-Y. HDOCK: a web server for protein-protein and protein-DNA/RNA docking based on a hybrid strategy. Nucleic Acids Res. 2017; 45: W365-W373.

[4] Li L, Xu J, Qiu G, Ying J, Du Z. Xiang T, et al. Epigenomic characterization of a p53-regulated 3p22.2 tumor suppressor that inhibits STAT3 phosphorylation via protein docking and is frequently methylated in esophageal and other carcinomas. Theranostics. 2018; 8: 61-77.

[5] Tang Z, Li C, Kang B, Gao G, Li C, Zhang Z. GEPIA: a web server for cancer and normal gene expression profiling and interactive analyses. Nucleic Acids Res. 2017; 45: W98-W102.
