# Supplementary material for: B-Myb accelerates colorectal cancer progression through reciprocal feed-forward transactivation of E2F2
Source: Oncogene. 2021 Jul 27;40(37):5613–25. doi: 10.1038/s41388-021-01961-9 (PMC8445821; doi:10.1038/s41388-021-01961-9)
Supplement: Supplementary file 2 — Supplementary Tables [file 41388_2021_1961_MOESM2_ESM.pdf]

Supplementary Tables

Supplementary Table S1. The clinical pathological information of Colorectal Cancer cDNA Array.

#	NO.	Gender	Stage
1	C1	Male	0
2	C1	Male	0
3	C2	Male	0
4	C3	Male	0
5	C4	Female	0
6	C5	Female	0
7	C6	Male	I
8	C7	Female	I
9	C8	Female	I
10	C9	Female	I
11	C10	Female	I
12	C11	Female	I
13	C12	Male	II
14	D1	Male	IIA
15	D2	Female	IIA
16	D3	Female	IIA
17	D4	Female	IIA
18	D5	Male	IIA
19	D6	Female	IIA
20	D7	Female	IIA
21	D8	Male	IIA
22	D9	Male	IIA
23	D10	Male	IIA
24	D11	Male	IIA
25	D12	Male	IIA
26	E1	Male	IIA
27	E2	Male	IIA
28	E3	Female	IIB
29	E4	Male	IIB
30	E5	Male	IIB
31	E6	Male	III
32	E7	Male	III
33	E8	Female	III
34	E9	Female	III
35	E10	Female	III
36	E11	Female	IIIA
37	E12	Female	IIIA
38	F1	Male	IIIA
39	F2	Female	IIIB
40	F3	Male	IIIB

41	F4	Female	IIIB
42	F5	Male	IIIC
43	F6	Male	IIIC
44	F7	Male	IIIC
45	F8	Male	IV
46	F9	Male	IV
47	F10	Female	IV
48	F11	Female	IV
49	F12	Female	IV

Supplementary Table S2. Immunohistochemistry analysis of B-Myb expression in colorectal cancer.

Pathological variables	Sample no.	B-Myb IHC staining (%)		*P* value
		Negative	Positive	
Normal tissues	10	5 (50.0)	5 (0)	**0.045***
Primary tumors	109	21 (19.3)	84 (80.7)	
**Stage**				**0.631**
Ⅰ＋Ⅱ	71	14 (19.7)	57 (80.3)	
Ⅲ＋Ⅳ	38	9 (23.7)	29 (76.3)	
**Lymph node status**				**0.604**
pN0	72	12 (16.7)	60 (83.3)	
pN1+	37	8 (21.6)	29 (78.4)	
**Grade**				**0.032***
1	32	3 (9.3)	29 (90.7)	
2	40	7 (17.5)	33 (83.5)	
3	21	8 (38.1)	13 (61.9)	
**Missing**	**15**			
**Tumour size**				**0.701**
T1-3	56	10 (17.9)	46 (82.1)	
T4	53	11 (20.8)	42 (79.2)	

***P < 0.05 (Chi-square tests)**

Supplementary Table S3. The clinical information of colorectal cancer tissue microarray.

#	position	sex	Age	Tissue	Pathological Diagnisis	Grade	Stage	TNM
1	1 A	F	40	Colon	Adenocarcinoma	1	IIB	T4N0M0
2	1 B	M	67	Colon	Mucinous adenocarcinoma	1	IIA	T3N0M0
3	1 C	M	73	Colon	Adenocarcinoma	1	IIA	T3N0M0
4	1 D	F	50	Colon	Adenocarcinoma	1	IIIC	T4N2M0
5	1 E	F	51	Colon	Adenocarcinoma	2	IIB	T4N0M0
6	1 F	M	36	Colon	Adenocarcinoma	1	IIB	T4N0M0
7	1 G	M	70	Colon	Adenocarcinoma	1	I	T2N0M0
8	1 H	M	54	Colon	Adenocarcinoma	1	IIIC	T4N2M0
9	1 I	M	31	Colon	Adenocarcinoma	1	IIB	T4N0M0
10	1 J	M	72	Colon	Adenocarcinoma	1	IIIB	T3N1M0
11	1 K	F	60	Colon	Adenocarcinoma	–	IIA	T3N0M0
12	1 L	F	55	Colon	Adenocarcinoma	1	IIA	T3N0M0
13	2 A	M	74	Colon	Adenocarcinoma	1	IIA	T3N0M0
14	2 B	M	63	Colon	Adenocarcinoma	1	IIA	T3N0M0
15	2 C	F	33	Colon	Adenocarcinoma	1	IIIC	T3N2M0
16	2 D	M	51	Colon	Adenocarcinoma	1	I	T2N0M0
17	2 E	F	49	Colon	Adenocarcinoma	1	IIIB	T4N1M0
18	2 F	M	77	Colon	Adenocarcinoma	1	IIB	T4N0M0
19	2 G	M	45	Colon	Mucinous adenocarcinoma	1	IIB	T4N0M0
0	2 H	M	79	Colon	Adenocarcinoma	1	IIB	T4N0M0
21	2 I	M	83	Colon	Adenocarcinoma	1	IIIB	T4N1M0
22	2 J	F	67	Colon	Adenocarcinoma	–	IIIB	T3N1M0
23	2 K	F	68	Colon	Mucinous adenocarcinoma	1	IIA	T3N0M0
24	2 L	M	64	Colon	Adenocarcinoma	1	IIA	T3N0M0
25	3 A	F	59	Colon	Mucinous adenocarcinoma	2	IIIB	T3N1M0
26	3 B	M	34	Colon	Mucinous adenocarcinoma	3	IIA	T3N0M0
27	3 C	M	58	Colon	Mucinous adenocarcinoma	1	IV	T4N1M1
28	3 D	M	57	Colon	Mucinous adenocarcinoma	2	IIIC	T3N2M0
29	3 E	F	65	Colon	Adenocarcinoma	1	IIB	T4N0M0
30	3 F	F	43	Colon	Adenocarcinoma	1	IIIB	T3N1M0
31	3 G	M	73	Colon	Adenocarcinoma	–	IIIC	T4N2M0
32	3 H	M	58	Colon	Adenocarcinoma	2	IIB	T4N0M0
33	3 I	F	82	Colon	Adenocarcinoma	2	IIB	T4N0M0
34	3 J	M	66	Colon	Mucinous adenocarcinoma	1	IIB	T4N0M0
35	3 K	F	52	Colon	Adenocarcinoma	2	IIA	T3N0M0
36	3 L	M	45	Colon	Adenocarcinoma	2	IIB	T4N0M0
37	4 A	F	71	Colon	Adenocarcinoma	1	IIB	T4N0M0
38	4 B	M	70	Colon	Adenocarcinoma	2	IIA	T3N0M0
39	4 C	M	77	Colon	Adenocarcinoma	2	IIB	T4N0M0
40	4 D	F	51	Colon	Adenocarcinoma	–	IIB	T4N0M0
41	4 E	M	63	Colon	Adenocarcinoma	2	IIB	T4N0M0

42	4 F	F	38	Colon	Adenocarcinoma	2	IIB	T4N0M0
43	4 G	F	58	Colon	Adenocarcinoma	2	IIB	T4N0M0
44	4 H	F	48	Colon	Adenocarcinoma	2	IIA	T3N0M0
45	4 I	M	63	Colon	Adenocarcinoma	2	IIA	T3N0M0
46	4 J	M	70	Colon	Adenocarcinoma	–	IIIB	T4N1M0
47	4 K	M	74	Colon	Adenocarcinoma	2	IIA	T3N0M0
48	4 L	M	39	Colon	Adenocarcinoma	2	IIIB	T4N1M0
49	5 A	F	41	Colon	Adenocarcinoma	1	IIA	T3N0M0
50	5 B	M	38	Colon	Adenocarcinoma	1	IIIB	T4N1M0
51	5 C	F	51	Colon	Adenocarcinoma	1	IIA	T3N0M0
52	5 D	M	67	Colon	Adenocarcinoma	1	IIA	T3N0M0
53	5 E	F	42	Colon	Adenocarcinoma	1	IIB	T4N0M0
54	5 F	M	51	Colon	Adenocarcinoma	3	IIA	T3N0M0
55	5 G	M	31	Colon	Adenocarcinoma	3	IIB	T4N0M0
56	5 H	F	68	Colon	Adenocarcinoma	2	IIA	T3N0M0
57	5 I	M	61	Colon	Adenocarcinoma	–	IIB	T4N0M0
58	5 J	M	72	Colon	Adenocarcinoma	2	IIA	T3N0M0
59	5 K	M	67	Colon	Adenocarcinoma	2	IIIC	T4N2M0
60	5 L	M	66	Colon	Adenocarcinoma	2	IIA	T3N0M0
61	6 A	F	70	Colon	Mucinous adenocarcinoma	2	II	T3N0M0
62	6 B	M	74	Colon	Mucinous adenocarcinoma	2	IIA	T3N0M0
63	6 C	F	76	Colon	Adenocarcinoma	–	I	T2N0M0
64	6 D	F	52	Colon	Adenocarcinoma	–	IIB	T4N0M0
65	6 E	F	60	Colon	Adenocarcinoma	–	IIIB	T3N1M0
66	6 F	F	47	Colon	Mucinous adenocarcinoma	3	IV	T4N1M1
67	6 G	M	75	Colon	Adenocarcinoma	2	IIIB	T3N1M0
68	6 H	F	77	Colon	Adenocarcinoma	2	IIA	T3N0M0
69	6 I	F	70	Colon	Adenocarcinoma	2	IIIC	T3N2M0
70	6 J	M	33	Colon	Adenocarcinoma	2	IIIA	T2N1M0
71	6 K	M	43	Colon	Adenocarcinoma	2	IIIB	T3N1M0
72	6 L	M	52	Colon	Adenocarcinoma	2	IV	T2N1M1
73	7 A	M	39	Colon	Adenocarcinoma	2	IIIC	T4N2M0
74	7 B	M	62	Colon	Adenocarcinoma	1	IIA	T3N0M0
75	7 C	M	77	Colon	Adenocarcinoma	1	IIA	T3N0M0
76	7 D	M	45	Colon	Adenocarcinoma	1	IIB	T4N0M0
77	7 E	F	52	Colon	Adenocarcinoma	–	IIA	T3N0M0
78	7 F	F	73	Colon	Adenocarcinoma	2	IIIC	T4N2M0
79	7 G	M	75	Colon	Adenocarcinoma	2	IIA	T3N0M0
80	7 H	M	47	Colon	Adenocarcinoma	–	I	T2N0M0
81	7 I	M	48	Colon	Adenocarcinoma	3	IIA	T3N0M0
82	7 J	M	41	Colon	Adenocarcinoma	3	IIB	T4N0M0
83	7 K	M	34	Colon	Adenocarcinoma	2	IIB	T4N0M0
84	7 L	M	79	Colon	Adenocarcinoma	2	IIA	T3N0M0
85	8 A	M	73	Colon	Adenocarcinoma	2	IIIC	T4N2M0
86	8 B	M	60	Colon	Adenocarcinoma	2	IIA	T3N0M0

87	8 C	M	47	Colon	Adenocarcinoma	–	IIB	T4N0M0
88	8 D	M	52	Colon	Adenocarcinoma	2	IIIB	T3N1M0
89	8 E	M	63	Colon	Adenocarcinoma	3	IV	T4N0M1
90	8 F	M	41	Colon	Adenocarcinoma	2	IIIB	T4N1M0
91	8 G	M	52	Colon	Adenocarcinoma	2	II	T4N0M0
92	8 H	M	70	Colon	Mucinous adenocarcinoma	2	IIB	T4N0M0
93	8 I	F	24	Colon	Adenocarcinoma	3	IV	T4N2M1
94	8 J	M	58	Colon	Adenocarcinoma	3	IIIB	T3N1M0
95	8 K	M	35	Colon	Adenocarcinoma	3	IIA	T3N0M0
96	8 L	M	71	Colon	Adenocarcinoma	3	IIB	T4N0M0
97	9 A	M	68	Colon	Adenocarcinoma	3	IV	T4N0M1
98	9 B	M	56	Colon	Mucinous adenocarcinoma	3	IV	T3N1M1
99	9 C	M	27	Colon	Mucinous adenocarcinoma	3	IIIC	T3N2M0
100	9 D	M	55	Colon	Adenocarcinoma	–	IIA	T3N0M0
101	9 E	M	48	Colon	Mucinous adenocarcinoma	3	IIA	T3N0M0
102	9 F	M	68	Colon	Adenocarcinoma	3	III	T3N1M0
103	9 G	M	37	Colon	Adenocarcinoma	3	IIB	T4N0M0
104	9 H	F	71	Colon	Adenocarcinoma	3	IIIB	T4N1M0
105	9 I	M	63	Colon	Adenocarcinoma	3	IIA	T3N0M0
106	9 J	F	49	Colon	Mucinous adenocarcinoma	3	IIB	T4N0M0
107	9 K	M	51	Colon	Mucinous adenocarcinoma	3	IIIB	T4N1M0
108	9 L	M	68	Colon	Adenocarcinoma	2	IIIB	T4N1M0
109	10 A	M	72	Colon	Adenocarcinoma	3	IIB	T4N0M0
110	10 B	F	34	Colon	Adenocarcinoma	–	IV	T4N1M1
111	10 C	M	31	Colon	Normal colonic tissue	–	–	–
112	10 D	M	28	Colon	Normal colonic tissue	–	–	–
113	10 E	M	32	Colon	Normal colonic tissue	–	–	–
114	10 F	M	34	Colon	Normal colonic tissue	–	–	–
115	10 G	M	32	Colon	Normal colonic tissue	–	–	–
116	10 H	M	37	Colon	Normal colonic tissue	–	–	–
117	10 I	M	32	Colon	Normal colonic tissue	–	–	–
118	10 J	M	41	Colon	Normal colonic tissue	–	–	–
119	10 K	M	32	Colon	Normal colonic tissue	–	–	–
120	10 L	M	34	Colon	Normal colonic tissue	–	–	–
121	10 M	–	–	–	Array marker	–	–	–

Supplementary Table S4. Antibodies used in the present study.

Protein name	Manufacture (cat. number)	Applications (working dilution)	Website Link
GAPDH	Xianzhi Bio (AB-P-R 001)	IB (1:5000)	http://www.goodhere.com/showproduct.asp?id=320&classid=34&nid=2
B-Myb	Santa Cruz (N-19):sc-724	IB (1:500)	http://www.scbt.com/datasheet-724-b-myb-n-19-antibody.html
B-Myb	Santa Cruz (H-115):sc-13028	IHC (1:50)	http://www.scbt.com/datasheet-13028-b-myb-h-115-antibody.html
E2F2	Santa Cruz (TFE-25):sc-9967	IB (1:500) IF (1:200)	https://www.scbt.com/scbt/zh/product/e2f-2-antibody-tfe-25?requestFrom=search
ERK	Cell Signaling Technology (4695S)	IB (1:2000)	http://www.cst-c.com.cn/products/4695.html
p-ERK	Cell Signaling Technology (4370S)	IB (1:2000)	http://www.cst-c.com.cn/products/4370.html
p-Akt	Ruiying Bio (RLP0006)	IB (1:500)	http://rlgene.com/showproduct.asp?/1_439
HA	Beyotime (AH158)	IF (1:300)	http://www.beyotime.com/product/AH158.htm
BrdU	Sigma (B2531)	IF (1:500)	http://www.sigmaaldrich.com/catalog/product/sigma/b2531?lang=zh&regio
pHH3	Cell Signaling Technology (9706)	IF (1:200)	https://www.cellsignal.com/products/primary-antibodies/phospho-histone-h3-ser10-6g3-mouse-mab/9706?N=4294956287&Ntt=Phospho+Histone+H3++ser10&fromPage=plp
IgG	Beyotime (A7016)	IP	https://www.beyotime.com/product/A7016.htm
Flag	Sigma (F1804-200UG)	IP	https://www.sigmaaldrich.com/catalog/product/sigma/f1804?lang=zh&region=CN
antiMouse secondary antibody	Abgent (ASS1007)	IB (1:5000)	http://www.abgent.com/products/ASS1007-Goat-Anti-Mouse-IgGHL-Human-ads-HRP-Secondary-Antibody
antiRabbit secondary antibody	Abgent (ASS1009)	IB (1:5000)	http://www.abgent.com/products/ASS1009-Goat-Anti-Rabbit-IgGHL-MouseHuman-ads-HRP-Secondary-Antibody
antiMouse seccondary antibody	Thermo (A-11031)	IF (1:1000)	https://www.thermofisher.com/antibody/product/Goat-anti-Mouse-IgG-H-L-Secondary-Antibody-Polyclonal/A-11031
antiRabbit seccondary antibody	Thermo (A-11034)	IF (1:1000)	https://www.thermofisher.com/antibody/product/Goat-anti-Rabbit-IgG-H-L-Secondary-Antibody-Polyclonal/A-11034

Supplementary Table S5. List of primer sequences used for qRT-PCR analyses.

Gene name	Primer name and sequences
GAPDH	F833: ACCTGACCTGCCGTCTAGAA
	R1060: TCCACCACCCTGTTGCTGTA
B-Myb	F484: AAGATGTTGCCAGGGAGGAC
	R679: TGGTCAGAAGACTTCCCTGG
ZNF473	F337: TGGACTTCACCTTGGGAGAC
	R535: TCAGTCACATCAGGGCTTGT
ESCO2	F1370: AGCAGAGTTTTGGGATGGGA
	R1599: CAGACAGGACACGAAATGCC
KIF11	F1110: GGAAGTGAAAACATTGGCCG
	R1299: ATGTTCTTGTACGCCCTCCA
NRP1	F1501: ACAGGTAGACTTGGGCCTTC
	R1694: CAACATCTGTGGGGTTGGTG
E2F1	F291: CCATCAGTACCTGGCCGAGAGC
	R494: CGCTTCTGCACCTTCAGCACCT
E2F2	F809: ACTCGGTATGACACTTCGCT
	R1036: TCTGGTGGGGTCTTCAAACA
E2F3	F409: GAGCTAGGAGAAAGCGGTCA
	R509: GGAGTTTTTGGACTATCTGGAC
E2F8	F74: CCACCACAGCAAATATCGTG
	R283: CTTTGGCCTCAGGTAATCCA
WISP2	F1025: GTCGCAGTCCACAAAACAGT
	R1184: GGTGGACCCAAGCTAAAGTG
DLC1	F606: AACTCCGTCATCAGCGTTTG
	R766: TCCATCCGTTTCAGCAGACT
KBTBD6	F988: GGAGAGTGAGCAGACAGTGT
	R1140: TTGGTCAGCAGCCCTTCTAA
IGFBP3	F613: TCTGATCCCAAGTTCCACCC
	R785: TCCATTTCTCTACGGCAGGG

Supplementary Table S6. Sequences of primers and siRNAs.

Primers for luciferase reporter constructions

Constructs	Methods	Primers and/or Enzymes used
E2F2-P1314	PCR based cloning	F1017: CTATCGATAGGTACC*GAGCTC*TGAAGGTTTTTGAATGAGGCAG *Sac I*
		R2329: GCTTACTTAGATCGC*AGATCT*AGCCGCCAATGGACGCCTG *Bgl II*
3×E2F reporter	PCR based cloning	PGL4.27-F-E2F: *TTTCGCGCTTAA*TTTCGCGCTTAATTTCGCGCTTAAAGATCTGGCC TCGGCGGCCAAG consensus E2F binding site
		PGL4.27 –R: GCTAGCGAGCTCAGGTACCGGC
B-Myb-P1064	PCR based cloning	B-Myb-F1085:CGG*GGTACC*CAGTCTAGGCAACAGCAACATAGCA *KpnI*
		B-Myb-R2148:CCG*CTCGAG*AGGCGTCAGCGTGTCAGCAGGT *XhoI*

Primers for mutant constructions

Name	Sequences
E2F2-P1314-MBS(1/2)m	Sense: 5'-GCCAGTGAAAAACAAGGGCTCCAGAGCTGGGACC-3'
	Antisense: 5'-AGGGGTTCTTCTACTCTCCCAAGCTGCCTGGG-3'
E2F2-P1314-EBS(1/2)m	Sense: 5'-AACTAAAAAAAAAAAAAAAAGAAGAAAAAAAAGAGGC-3'
	Antisense: 5'-CCAAACGTCGTGCCGCGAAATTCGGATCTCCCGCG-3'
E2F2-P1314-EBS(1/2)m+MBS(1/2)m	Sense: 5'-AACTAAAAAAAAAAAAAAAAGAAGAAAAAAAAGAGGC-3'
	Antisense: 5'-CCAAACGTCGTGCCGCGAAATTCGGATCTCCCGCG-3'
mutant 3xE2F reporter	Sense:5'-TTTCGATCTTAATTTCGATCTTAATTTCGATCTTAAAGATCTGGCCTCGGCGGC CAAG-3'
	Antisense: 5'-GCTAGCGAGCTCAGGTACCGGC TT-3'
B-Myb-P1064-MBS(1/2)m	Sense: 5'-CCTGCGAGCGAGGAGCGCGGGACCTGCTGA-3'
	Antisense: 5'-CCAGTCAGTCGCCGCCGGCGCGGGTTGAAG-3'
B-Myb-P1064-EBSm	Sense:5'-TATGGAGATAGAAAAGTGCTTCAACCCGCGCCGGCGGCGA-3'
	Antisense: 5'-CAAGCGCGTCGGCGCCGCCCAGTCCT-3'
B-Myb-P1064-EBSm+MBS(1/2)m	Sense:5'-TATGGAGATAGAAAAGTGCTTCAACCCGCGCCGGCGGCGA-3'
	Antisense: 5'-CAAGCGCGTCGGCGCCGCCCAGTCCT-3'

Primers for truncated constructions

Name	Sequences
pCDH-puro-HA-E2F2	Sense: 5'-TACCCAGACGTCCCAGACTACGCTCTGCAAGGGCCCCGGGCCT-3'
	Antisense: 5'-CATGGTGGCAGATCCGAGCTCG-3'
pCDH-puro-HA-E2F2△MB	Sense: 5'-GTTGGCCTTGTCCTCAGTCAGGTG-3'
	Antisense: 5'-CTCCCCTCTACCTCCACCCTCTG-3'
pCDH-puro-HA-E2F2(1-244)	Sense: 5'-GTTGGCCTTGTCCTCAGTCAGGTG-3'
	Antisense: 5'-TGAGATCCACTAGTAACGGCCGCC-3'
pCDH-puro-HA-E2F2(245-437)	Sense: 5'-CATGGTGGCAGATCCGAGCTCG-3'
	Antisense: 5'-AAGAGGCTGGCCTATGTGACTTACCAG-3'

siRNA sequences.

Name	Sequences
Negative control siRNA	Sense: 5'-UUCUCCGAACGUGUCACGUTT-3'
	Antisense: 5'-ACGUGACACGUUCGGAGAATT-3'
B-Myb siRNA	Sense: 5'-CAGACAAUGCUGUGAAGAATT-3'
	Antisense: 5'-UUCUUCACAGCAUUGUCUGTT-3'
E2F2 siRNA	Sense: 5'-GACUCGGUAUGACACUUCGTT-3'
	Antisense: 5'-CGAAGUGUCAUACCGAGUC TT-3'
