# Supplementary material for: B-Myb accelerates colorectal cancer progression through reciprocal feed-forward transactivation of E2F2
Source: Oncogene. 2021 Jul 27;40(37):5613–25. doi: 10.1038/s41388-021-01961-9 (PMC8445821; doi:10.1038/s41388-021-01961-9)
Supplement: Supplementary file 3 — Supplementary Figure Legends [file 41388_2021_1961_MOESM3_ESM.pdf]

Supplementary Figures

Supplementary Figure 1. GO and Pathway analyses on B-Myb-regulated genes.

(a) GO enrichment and (b) KEGG analyses were performed based on the differentially expressed genes between the stable B-Myb knockdown (shB-Myb) and its control (shNC) HCT116 cells.

Supplementary Figure 2. GSEA analysis on B-Myb-regulated genes.

Top enriched gene sets by GSEA analysis. (a) RB_P107 gene signatures, (b) MYC gene signatures, (c) E2F3 gene signatures, (d) SRC gene signatures and (e) PTEN gene signatures.

Supplementary Figure 3. E2F2 is required for B-Myb-induced phenotype in RKO cells.

(a) siRNA mediated silencing of B-Myb and E2F2. Stable B-Myb overexpression (LV-B-Myb, upper panel) RKO cells were transiently transfected with negative control siRNA, B-Myb siRNA or E2F2 siRNA. Stable B-Myb knockdown (shB-Myb, lower panel) RKO cells were transiently transfected with pcDNA3.0 empty vector, LV203-B-Myb-Flag and pcDNA3.1-E2F2 expression constructs. Twenty-four hours after transfection, qRT-PCR was performed to determine B-Myb and E2F2 mRNA expression. (b-d) E2F2 is required for B-Myb-induced cell proliferation and motility. Cells were transiently transfected as described in (a), and cell proliferation and motility were detected by CCK8 assay (b), wound healing assay (c) and transwell migration assay (d). Representative images (×200) and quantification results were indicated for each assay. Data represent the mean ± SD. All experiments were performed in triplicates. * $p < 0.05$, ** $p < 0.01$, *** $p < 0.001$.

Supplementary Figure 4. E2F2 is required for B-Myb-induced motility in HCT116 cells.

Stable B-Myb overexpression (LV-B-Myb) or Stable B-Myb knockdown (shB-Myb) HCT116 cells were established as in Figure 8a. Cells were then transiently transfected as described in Figure 8a, and cell motility were determined wound healing assay (a) and transwell migration assay (b) as described in Supplementary Figure 3c-d.
