# Supplementary figures and images for: B-Myb accelerates colorectal cancer progression through reciprocal feed-forward transactivation of E2F2
Source: Oncogene. 2021 Jul 27;40(37):5613–25. doi: 10.1038/s41388-021-01961-9 (PMC8445821; doi:10.1038/s41388-021-01961-9)

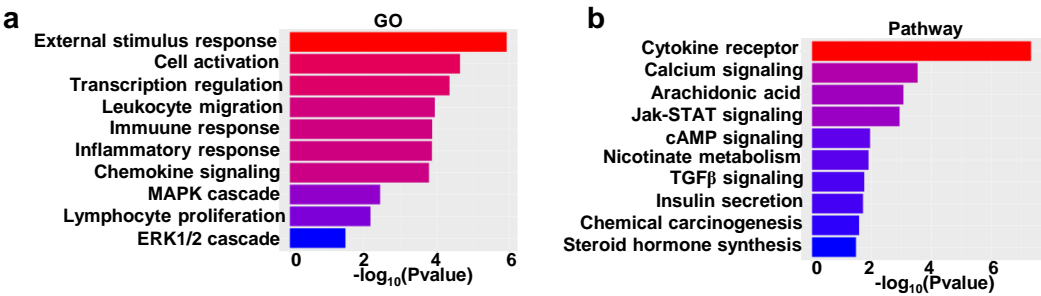

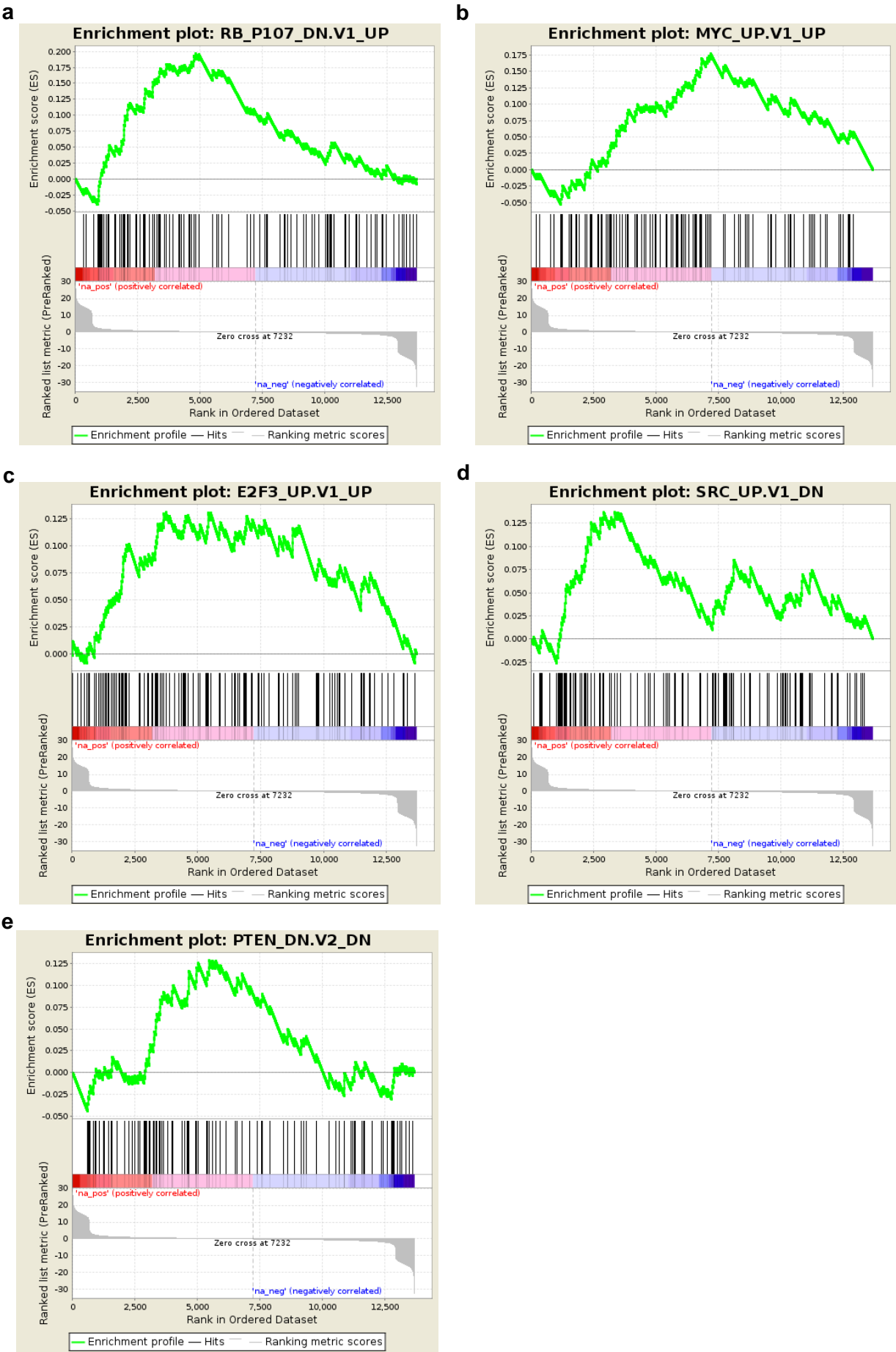

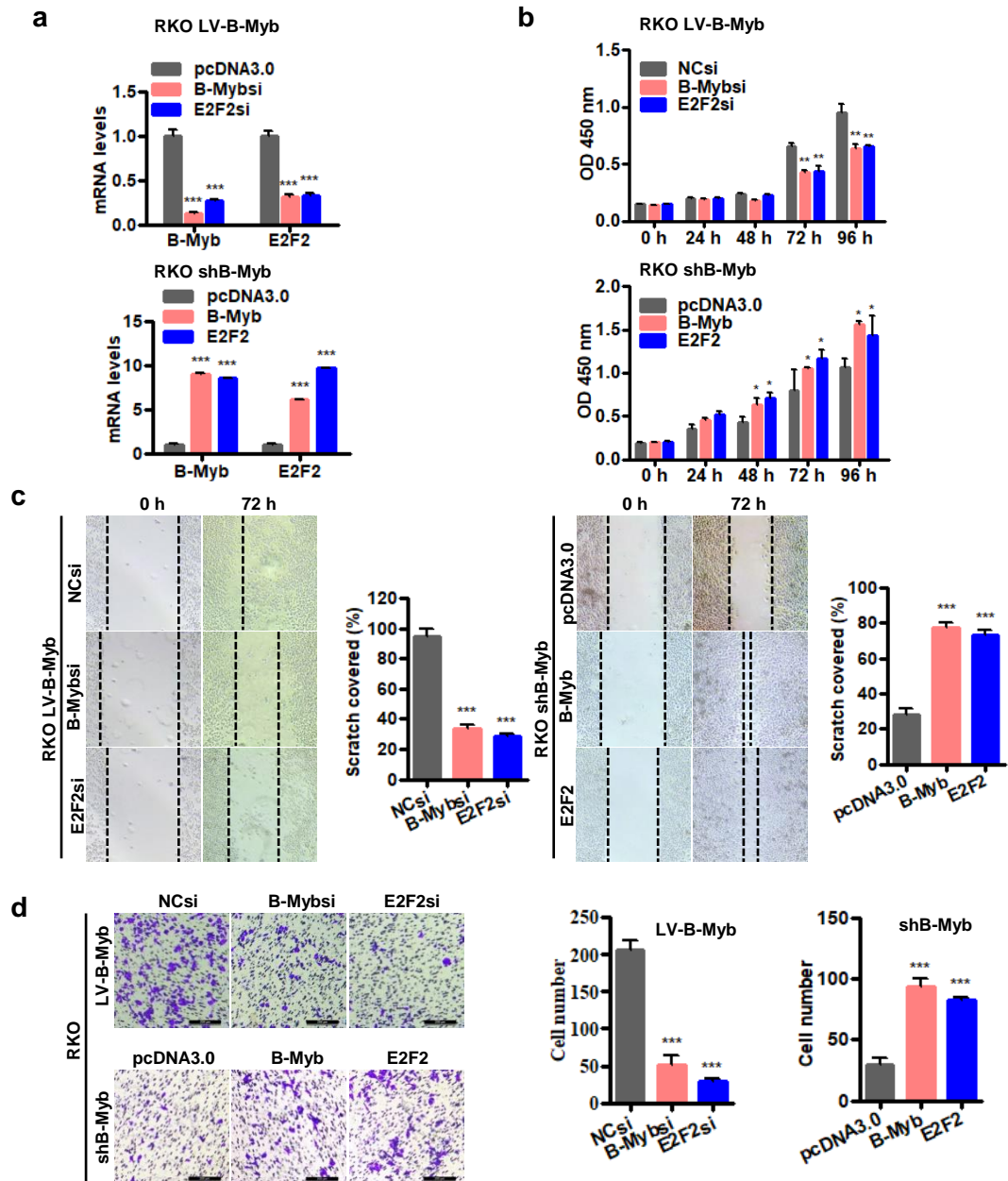

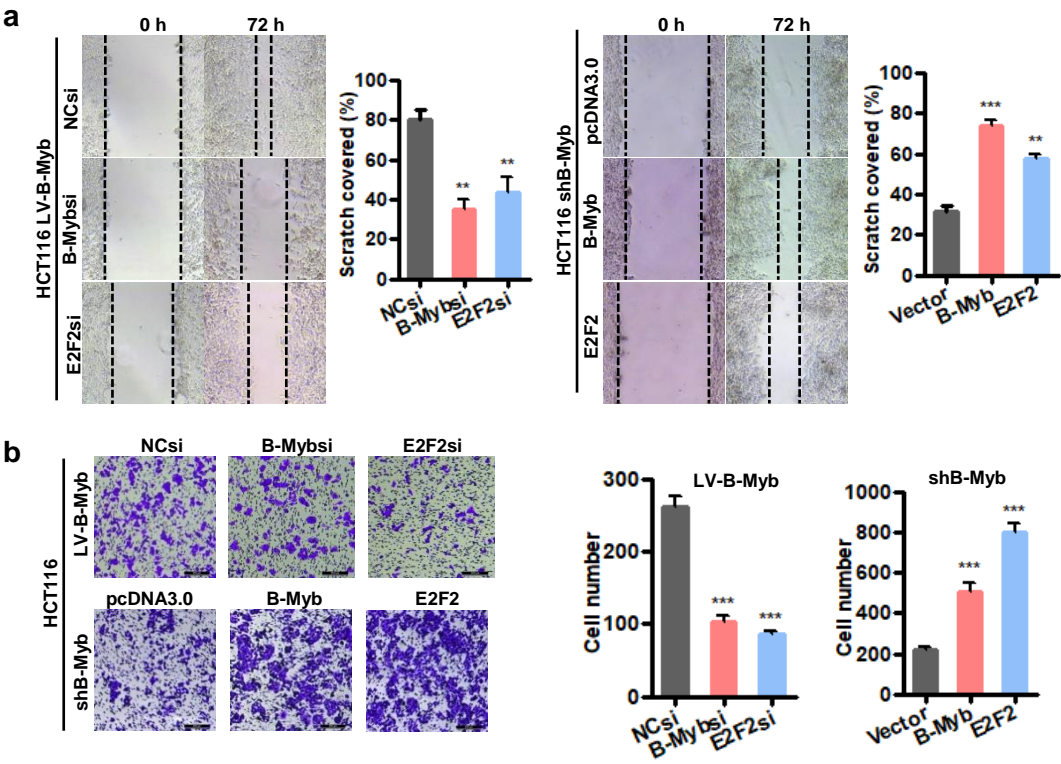

Supplement: Supplementary file 4 — Supplementary Figure 1-4 [file 41388_2021_1961_MOESM4_ESM.pdf]
